# Impact of Positive Lymph Nodes after Systematic Perihilar Lymphadenectomy in Colorectal Liver Metastases

**DOI:** 10.3390/jcm13175301

**Published:** 2024-09-06

**Authors:** Gabriel F. Hess, Noa L. E. Aegerter, Jasmin Zeindler, Jürg Vosbeck, Kerstin J. Neuschütz, Philip C. Müller, Simone Muenst, Silvio Däster, Martin Bolli, Otto Kollmar, Savas D. Soysal

**Affiliations:** 1 Clarunis, University Digestive Health Care Centre Basel, 4002 Basel, Switzerlandnoa.aegerter@usb.ch (N.L.E.A.);; 2 Institute of Medical Genetics and Pathology, University Hospital Basel, Schönbeinstrasse 40, 4031 Basel, Switzerland; juerg.vosbeck@usb.ch (J.V.); simone.muenst@usb.ch (S.M.); 3 Faculty of Medicine, University of Basel, Klingelbergstrasse 61, 4056 Basel, Switzerland; silvio.daester@unibas.ch; 4 Cantonal Hospital of Lucerne, Spitalstrasse, 6000 Luzern, Switzerland

**Keywords:** colorectal liver metastases, liver surgery, perihilar lymph nodes

## Abstract

**Background:** 25 to 50% of patients suffering from colorectal cancer develop liver metastases. The incidence of regional lymph node (LN) metastases within the liver is up to 14%. The need for perihilar lymph node dissection (LND) is still a controversial topic in patients with colorectal liver metastases (CRLM). This study investigates the role of perihilar LND in patients with CRLM. **Methods:** For this retrospective study, patients undergoing surgery for CRLM at the University Hospital Basel between May 2009 and December 2021 were included. In patients with perihilar LND, LN were stained for CK22 and examined for single tumour cells (<0.2 mm), micro- (0.2–2 mm), and macro-metastases (>2 mm). **Results:** 112 patients undergoing surgery for CRLM were included. 54 patients underwent LND, 58/112 underwent liver resection only (LR). 3/54 (5.6%) showed perihilar LN metastases in preoperative imaging, and in 10/54 (18.5%), micro-metastases could be proven after CK22 staining. Overall complications were similar in both groups (LND: 46, 85.2%; LR: 48, 79.3%; *p* = 0.800). The rate of major complications was higher in the LND group (LND: 22, 40.7%; LR: 18, 31%, *p* = 0.002). Median recurrence-free survival (RFS) (LND: 10 months; LR: 15 months, *p* = 0.076) and overall survival (OS) were similar (LND: 49 months; LR: 60 months, *p* = 0.959). **Conclusion:** Preoperative imaging is not sensitive enough to detect perihilar LN metastases. Perihilar LND enables precise tumour staging by detecting more lymph node metastases, especially through CK22 staining. However, perihilar LND does not influence oncologic outcomes in patients with CRLM.

## 1. Introduction

Colorectal cancer (CRC) is the second most common cause of cancer-related death in the US, and the incidence of patients diagnosed with CRC has increased in the last few years [1,2]. The liver is the most common site for metastases from colon cancer [3]. Approximately 25 to 50% of patients suffering from CRC develop liver metastases [4].

The only potentially curative therapeutic option for colorectal liver metastases (CRLM) is liver resection, which has recently been performed more frequently [5,6]. However, up to 80 percent of patients with CRLM are not operable. In those cases, five-year survival is near zero compared to a five-year survival rate of up to 50% in patients who can be treated with surgery [7].

Standard preoperative imaging for staging of CRLM consists of CT scans and MRI, whereby MRI detects small lesions more reliably [4]. Some assume that MRI is also the best type of imaging to detect small LN metastases [8]. However, current studies report inadequate LN staging by preoperative imaging compared to the histopathological findings after removing LNs [9,10].

**Figure 1:**
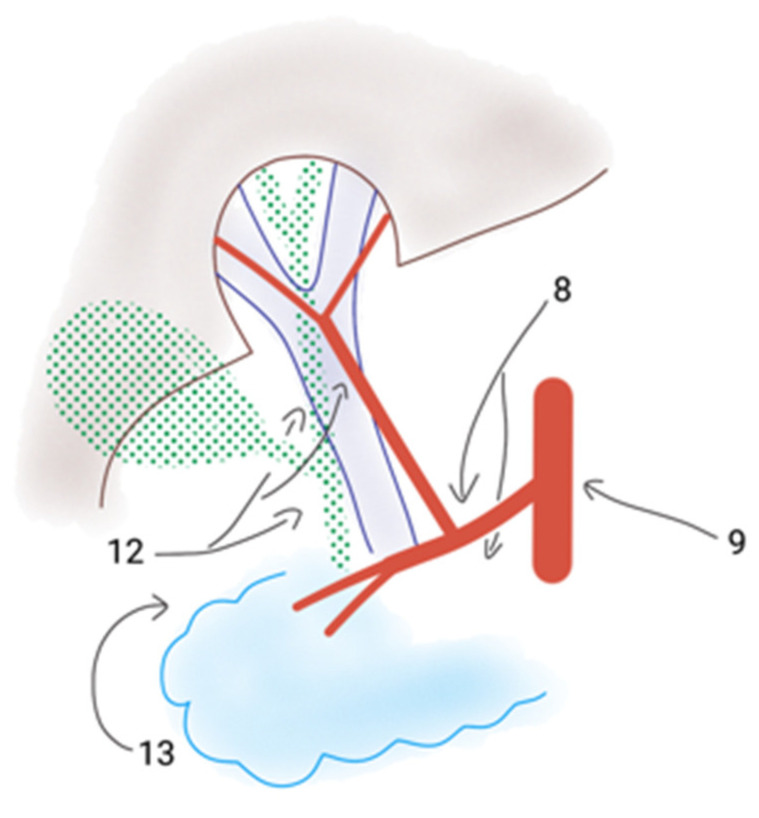
Lymphatic drainage of the liver hilus and the LN stations. Station 8: LNs along the common hepatic artery; station 9: LNs along the celiac trunk; station 12: LNs in the hepatoduodenal ligament along the hepatic artery, the bile duct, and behind the portal vein; station 13: LNs on the posterior surface of the pancreatic head.

According to Bork et al., it is hypothesized that perihepatic LN metastases from CRC occur through lymphatic vessel invasion by the metastases as well as by the primary tumour [11]. The lymphatic drainage of the liver follows superficial and deep lymph vessels into LN and further lymphatic vessels along the portal vein and the common hepatic artery, the superior mesenteric artery, and the coeliac trunk to the paraaortic LN [11]. For comparability, perihilar LNs are often classified according to the Japanese classification system [11,12]. Important LNs in the hepatoduodenal ligament and around the hepatic vasculature are stations 8, 9, 12, and 13 [11] (Figure 1).

Figure 1 shows the localisation of perihilar LN with the numbers indicating the stations according to the Japanese LN classification [12].

The incidence of regional LN metastases around the liver in patients with CRLM is reported up to 14% after histologic examination [13]. The discussion of whether regional lymph node dissection (LND) should be performed in cases of operable CRLM remains controversial. For example, the ESMO guidelines do not specify whether perihilar LND should be performed [14]. However, a complete resection of all tumour masses is recommended, as it serves as the best curative treatment approach [14]. According to Koti et al. and Pindak et al., LND is not recommended as a routine procedure since LND has not been shown to benefit patient survival. However, they mention a lack of prospective studies [15,16]. On the other hand, some studies mention that suspicious LNs should be resected [9]. Some only favour only systematic LND, as it provides accurate staging [17,18]. Liu et al. even propose hilar LND, which might offer a complete curative treatment option [19].

However, some studies have only examined the outcomes after selective, not after systematic, LND. Therefore, results can also be inaccurate [16]. Furthermore, to our knowledge, only one other study from Bennett et al. used the CK22 staging to examine the harvested LNs in detail [20].

Another important point when considering LND is the possible increase in morbidity through extensive harvesting of LNs [19,21,22].

Therefore, in this study, the resected LNs were examined with immunohistochemical staining to gain a detailed analysis of the hilar LNs that might contain tumour cells. Furthermore, we investigated the role of systematic perihilar LND compared to liver resection only (LR) in patients with CRLM and its impact on morbidity, overall survival, and recurrence to aid decision-making when it comes to perihilar LND in patients with CRLM.

## 2. Materials and Methods

### 2.1. Study Design

The University Hospital Basel is a tertiary centre for hepato-biliary surgery in Switzerland with approximately 100 liver resections per year. In this retrospective single-centre analysis, all patients undergoing liver surgery for CRLM between May 2009 and December 2021 were included. The patients were stratified into two groups. As no systematic perihilar LND was performed between 2009 and 2018 in patients undergoing liver resection for CRLM, these patients served as the study’s control group (LR). From 2019 until 2021, all patients undergoing liver resection for CRLM additionally received systematic perihilar LND and were included in the LND group. Ethical approval was granted by the ethics commission of Northwest Switzerland with the registration number EKNZ 2020–00076 on the 16th of April 2020.

All openly performed liver resections due to CRLM were included, as were patients over 18 years old and with available written informed consent. Patients with and without preoperative chemotherapy were included. Exclusion criteria were other entities of liver tumours and emergency surgery.

**Figure 2:**
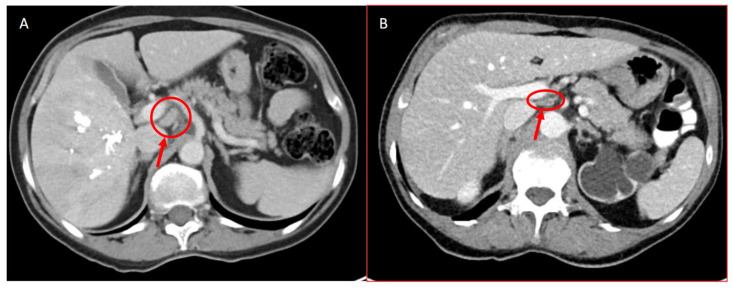
CT scans of patients with LN metastasis. (**A**) shows a CT scan of a LN+ patient with an already on-imaging suspicious LN (red arrow). (**B**) shows a CT scan of a LN+ patient where the LNs on imaging were not suspicious (red arrow).

Preoperative imaging, such as CT scans or MRI, was examined for abnormal LNs (Figure 2). LNs were classified as abnormal if they were either enlarged or increased in number. Qualified radiologists assessed all preoperative imaging.

### 2.2. Data Collection

Data was extracted retrospectively from the dedicated electronic database (ISMED) by trained contributors with regular auditing and guidance by the principal investigator from a prospective consecutive institutional database (Microsoft Office created ACCESS-database, version 14.0.7015. 1000, Office 2013; Microsoft Corporation, Redmond, WA, USA). Patient records of hospitalisation, including relevant clinical follow-up information concerning recurrence, surgery procedures, or death were compiled.

Complications were categorised according to the Clavien–Dindo classification [23]. Major complications were defined as complications over grade II [23]. Early complications were defined as complications within 30 days after the surgery, and late complications were defined as complications later than 30 days. Follow-up data was collected through the patients’ re-admission and aftercare via regular imaging with CT scans. As patient data were collected retrospectively, no standard follow-up appointments were conducted.

Reporting followed the Strengthening the Reporting of Observational Studies in Epidemiology (STROBE) reporting guideline where appropriate [24].

### 2.3. Lymph Node Dissection

According to the Japanese LN classification, perihilar LND was defined as the resection of LN stations 8 and 12 [12]. In the LND group, the aim of the surgery was to dissect stations 8 and 12 as well as additional LNs if they were found to be suspicious intraoperatively. All patients with no perihilar or other LNs removed, as described by the Japanese LN classification, were assigned to the LR group [12].

### 2.4. Immunohistochemistry

**Figure 3:**
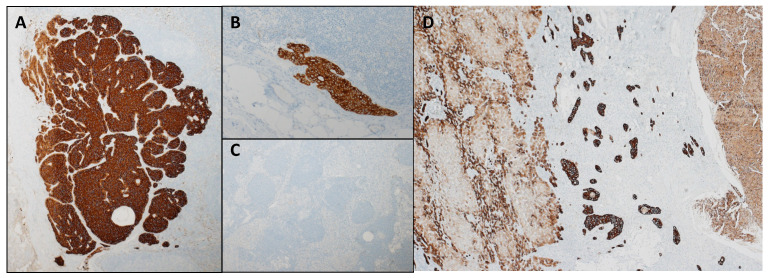
LN and CRLM with CK22 staining (Ventana). Lymph nodes with (**A**): Micro metastasis (0.2–2 mm, magnification 200 µm), (**B**): isolated tumour cells (<0.2 mm, magnification 100 µm), (**C**): normal lymphatic tissue. CK22 immunohistochemical staining, magnification 200 µm. (**D**): CRLM stained with CK22 for comparison (Lighter brown left of centre: hepatic parenchyma. Dark brown centre: Viable tumour cells. Lighter brown right of centre: necrotic tumour).

The LNs from the LND group were immunohistochemically stained with CK22 (Pancytokeratin Cocktail) CK22 is the standard pan-cytokeratin marker used at the Pathology Department of the University Hospital Basel. Other alternative markers would be Lu5, MNF116, or AE1/AE3, which could equally be used. The stained LNs were histologically examined for single cells (<0.2 mm), micro- (0.2–2 mm), and macro-metastases (>2 mm) (Figure 3).

Immunohistochemical staining was performed using 4-µm sections of the paraffin blocks. LNs were stained with CK22 antibody (Ventana). Immunohistochemistry was evaluated by a medical student [NLEA] and a surgical registrar [GFH] and supervised by two senior consultant pathologists [JV, SM].

**Figure 4:**
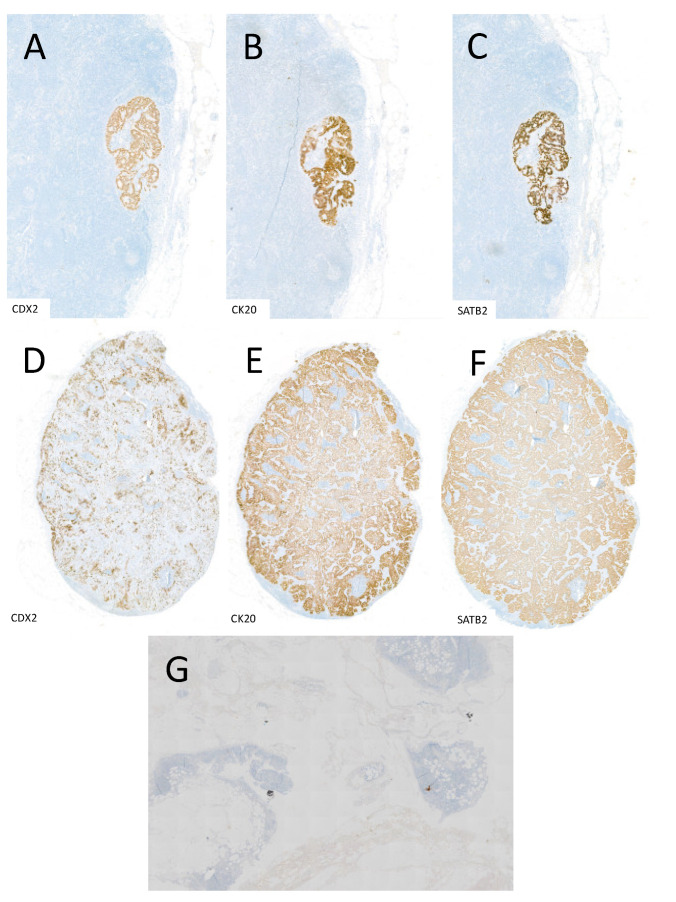
(**A**–**C**) display a LN with micrometastasis in a CDX2, CK20, and SATB2 stain. (**D**–**F**) display an LN with a macrometastasis in a CDX2, CK20, and SATB2 stain. (**G**) displays an image of a single tumour cell in CDX2 stain, which is not detectable except for the CK22 stain.

As a comparison to the CK22 stain, the resected LN metastases were also stained with CDX2, CK20, and SATB2 (Figure 4). CDX2, CK20, and SATB2 are all stains specific for CRC.

### 2.5. Outcomes

The primary endpoint of this study was to investigate the OS and RFS in patients with CRLM. Secondary endpoints included the impact of perihilar LND on postoperative complications as well as the accuracy of detecting LN metastases through imaging, routine pathological examination, and CK22-staining.

### 2.6. Statistical Analysis

Descriptive statistics were used to summarize patient, treatment, and disease characteristics. Data are shown as median or mean with the corresponding range. The significance of observed differences between the two groups was calculated using the Mann–Whitney U or the Fisher test as appropriate. As the data were non-normally distributed, the Mann–Whitney U test was used for continuous variables, and the Fisher test was used for categorical data. Statistical analyses were calculated using Prism 10 Version 10.0.0 (Graphpad Software, Inc., La Jolla, CA, USA).

## 3. Results

### 3.1. Patient Characteristics

A total of 112 patients were included. Of those, 54 patients underwent liver resection with LND, and 58 patients underwent liver resection only. Eleven patients of the LND group (20.4%) and 16 patients of the LR group (27.6%) had prior liver resection.

No significant difference regarding previous systemic therapy (LND: 38, 70.4%; LR: 35, 60.3%, *p* = 0.323) or previous surgery for CRC (LND: 36, 66.7%, LR: 38, 65.5%; *p* > 0.999) was found in the LND and LR patients.

Comorbidities such as hepatitis B, diabetes, cardiac and neurological history, or COPD occurred with similar frequencies in both groups. The detailed patient characteristics can be found in Table 1.

### 3.2. Perioperative Findings

Liver metastases were significantly larger in the LND group (LND 3.07 cm; LR 2.1 cm; *p* = 0.002), as shown in Table 2. Most patients in both groups had singular CRLM (LND: 18, 33.3%; LR: 18, 31%; *p* = 0.841). In the LND group, significantly more patients underwent anatomic liver resection than in the LR group, where non-anatomic liver resection was more often performed (LND 33, 61.1%; LR: 12, 20.7%; *p* < 0.001). The LND patients underwent significantly more hepatectomies (LND: 34, 62.96%; LR: 8, 13.8%; *p* < 0.001). The blood loss in the LND group was significantly higher than in the LR group (LND: 641 mL (50–2000); LR: 222 mL (0–1600); *p* < 0.001).

Atypical liver resections were performed more often in the LR group (LND: 5, 9.3%; LR 34, 58.6%, *p* < 0.001), while in the LND group, more than half of the patients underwent major hepatectomies (LND: 34, 62.96%; LR: 8, 13.8%, *p* < 0.001).

There was no significant difference regarding operation time (LND: 180 min, LR: 195 min, *p* = 0.064), but intraoperative blood loss was significantly higher in the LND patients (LND: 641 mL, LR 222 mL, *p* < 0.001).

### 3.3. Postoperative Complications

In the LND group, 46 patients (85.2%) had at least one postoperative complication compared to 48 (79.3%, *p* = 0.800) in the LR group. Major complications occurred in 22 (40.7%) patients in the LND group and 18 (31%) patients in the LR group, which was found to be significantly different (*p* = 0.0023).

When comparing the postoperative outcomes of patients who underwent hemihepatectomies, 14 out of 34 patients (41.2%) in the LND group had major complications, and 7 out of 8 (87.5%) in the LR group, which is a significant difference (*p* = 0.045).

In the LND group, two patients died postoperatively. One patient suffered from a severe ARDS (acute respiratory distress syndrome) two days after the surgery due to aspiration, which evolved into multi-organ failure. The other patient was readmitted two and a half months after the operation due to a neutropenic fever and a toxic megacolon and subsequently died.

In the LR group, one patient died postoperatively. Three days after the operation, the patient presented symptoms suspicious of a pulmonary embolism and required resuscitation, which was unsuccessful.

The ICU stay was significantly shorter in LR patients than in the LND group (LND: 2, LR: 1, *p* < 0.0001). Postoperative stays were similar in both groups (LND: 10, LR: 9, *p* = 0.435). This data is shown in Table 3.

### 3.4. LN Findings

Table 4 displays detailed results for the LND group. LND patients are further subdivided in groups with positive lymph nodes (LN+) and no LN metastases (LN−). The false-negative rate for the preoperative imaging in patients undergoing LND was 14.8%, and the false-positive rate was 1.85%.

In the LND group, most of the patients underwent resection for LN stations 8, 9, and 12 with additional resection, when they were assessed as suspicious intraoperatively or on preoperative imaging (LN+: 6, 60%; LN−: 23, 52.3%; *p* = 0.736).

Of the 54 patients who underwent LND, 3/54 (5.6%) showed suspicious LNs on preoperative imaging. After LND, routine pathological examination without a special stain detected 6 (11.1%, *p* = 0.270) patients with LN metastases. After staining all resected LNs with a cytokeratin antibody, ten patients with micrometastases or isolated tumour cells were found, significantly more than detected by preoperative imaging (18.5%, *p* = 0.029).

The resected LNs were additionally stained with specific tumour markers for CRC (Figure 4). Micro and macrometastases could be portrayed equally in the CK22 as well as the other CRC-specific stains. However, single tumour cells were only found by administrating CK22.

**Figure 5:**
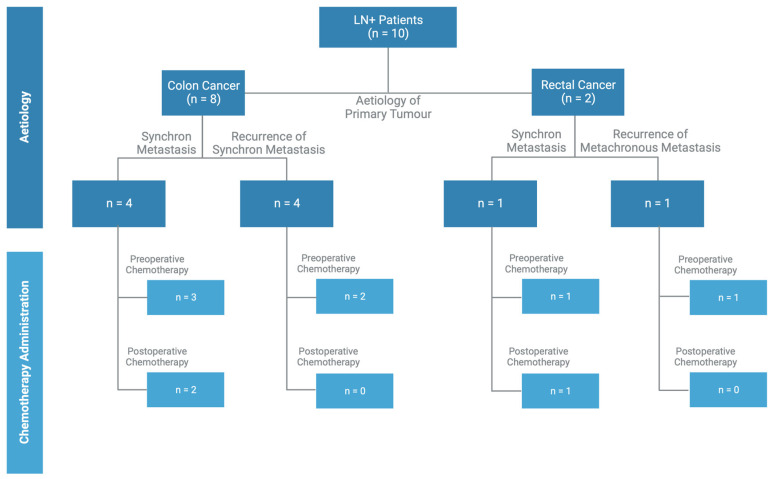
LN+ Findings. Aetiology of the primary tumour and display of postoperative treatments after liver resection in the LN+ group.

The results of the LN+ data show no clear administration pattern of pre- and postoperative chemotherapy (Figure 5).

### 3.5. Recurrence Rates

The mean follow-up period of the LND group was 24 months versus 34.5 months in the LR group (*p* = 0.006).

Administration of adjuvant systemic treatment was similar in both groups (LND 30, 55.6%; LR: 24, 41.4%; *p* = 0.185), as displayed in Table 5. Additional local therapy was present more often in the LND group (LND: 28, 51.9%; LR 10, 17.2%, *p* < 0.0001).

**Figure 6:**
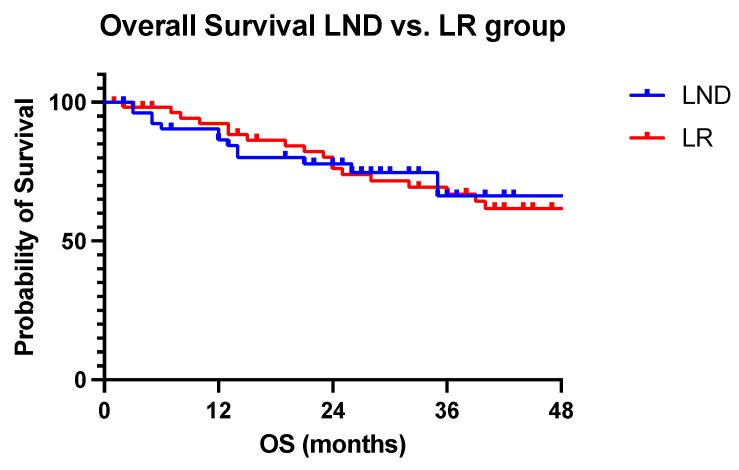
Overall Survival of LND vs. LR. Overall survival in the LND and LR patients. Log-rank, one-sided *p* (0.775).

**Figure 7:**
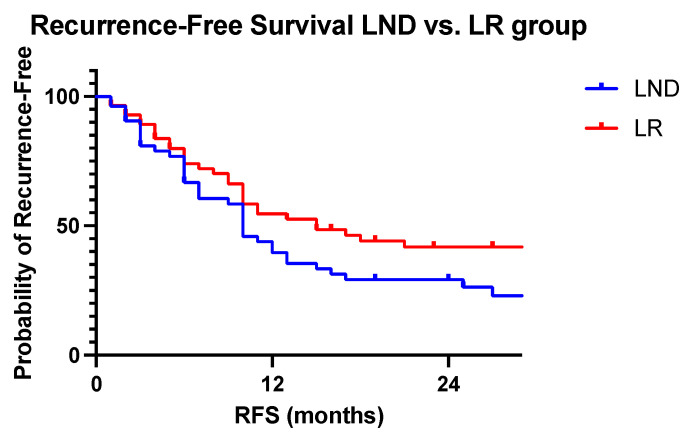
Recurrence-Free Survival. Recurrence-free survival in LND and LR patients. Kaplan–Meier for recurrence-free survival (RFS). For each patient not known to have died, RFS is censored at the time of the last date known to be alive; Log-rank, one-sided *p* (0.076).

Median overall survival was 49 months in the LND group and 60 in the LR group (*p* = 0.959). Median RFS was ten months in the LND group and 15 months in the LR group (*p* = 0.076). OS and RFS are displayed in Figure 6 and Figure 7.

## 4. Discussion

According to the results of this study, a systematically performed perihilar LND cannot be recommended to increase RFS or OS in patients with CRLM. However, an important number of patients with LN metastases could have only been detected through LND. Performing LND in cases with suspicious LNs pre- or intraoperatively is a reasonable approach.

Furthermore, preoperative imaging has been found to be not accurate enough to detect perihilar LN metastases.

Nevertheless, in this cohort, a significantly increased rate of major complications was observed in the LND group compared to the LR group.

The results of this study have shown that perihilar LND does not benefit RFS or OS in patients with CRLM. These findings align with those of Koti et al., who also found that routine LND does not increase recurrence-free survival in patients with CRLM [16]. Furthermore, Gurusamy et al. found that routine LND and selective LND ensure no survival benefit, irrespective of whether the positive LNs were detected through imaging or surgical LN removal [25].

On the other hand, Adam et al. vouch for LND in patients with isolated pedicular LN with a promising preoperative response to systemic chemotherapy, and Hodgson et al., as well as Liu et al., proposed LND in patients presenting with radiologically or clinically suspicious LNs [9,19,26].

Additionally, other studies have shown patients with positive perihilar LNs have worse overall survival, which underlines that accurate staging is indispensable for a curative treatment regimen [8,9,19].

To date, optimal treatment for patients with CRLM consists of surgical resection, often followed by adjuvant chemotherapy [27]. However, it is not conclusively defined whether adjuvant chemotherapy should be administered in patients with CRLM [14]. According to the ESMO guidelines, neoadjuvant chemotherapy can be omitted in cases of favourable disease [14]. Nevertheless, it seems difficult to provide patients with CRLM with the correct systemic treatment without proper staging.

Therefore, prospective randomised controlled trials are necessary for a precise recommendation concerning LND in patients with CRLM and to define the treatment of choice in patients with CRLM, especially those with positive LNs.

As others have mentioned, preoperative imaging does not seem accurate enough to detect LN metastases [9]. This is supported by the results of this study, which showed that out of the 54 patients undergoing LND, preoperative imaging missed LN metastases in eight (14.8%) of those patients.

The results of our study demonstrate that a significant number of LN metastases would have been missed without the dissection of the perihilar LN.

Moreover, four more LN+ patients have been detected through the administration of CK22-staining only [20].

A possible cause for the discrepancy between routine pathological findings and those with CK22-staining is that micro-metastases or single tumour cells are highlighted, which are too small to be detected by preoperative imaging and difficult to identify through routine pathological examination. Therefore, we recommend the use of CK22-stain when aiming for accurate staging of patients with CRLM.

Hodgson et al. reported a 100% positive predictive value when using PET to detect LN metastasis [9]. Zhu et al. developed a promising model with MR imaging to detect LN metastases [8]. However, further randomized controlled trials are needed to validate whether PET scans or models using clinical and MRI parameters can improve preoperative diagnosis of LN metastasis and, therefore, allow a better patient selection, which is also found by Martin et al. [4].

One crucial aspect to consider performing a perihilar LND are the possible complications. Perihilar LND, such as portal LND, bears the risk of common bile duct devascularization, damage to the surrounding vessels, postoperative lymphatic leakage, pancreatitis, and pancreatic fistula. Further, LND is claimed to prolong operating time [19,21,22]. However, Adam et al. consider LND a safe procedure with a 60-day mortality rate of zero in the group with regional LN metastases and 1% in the group without regional metastases [26].

When comparing the morbidity of hilar LND in this cohort, the LND group showed a significantly longer ICU stay and a significantly higher rate of major complications. Nonetheless, the LND group underwent significantly more hepatectomies than the LR group. This can be a reason why the complication rate is higher in patients after LND, as more extensive liver surgery elevates the risk of complications [28]. Moreover, when comparing the hepatectomies only in both groups, the complication rate is more than two-fold in the LR group than in the LND group. This is another indicator that the extent of the operation might be the reason for a higher complication rate in the LND group and not LND itself.

These findings differ from the study by Pindak et al., who found no significant difference in postoperative complications between the patients without LND and the selective LND group [15] and those of Adam et al. and Ercolani et al. [13,26].

Although the results of the comparison between the LND and LR groups can possibly lead to the conclusion that LND increases the risk of major complications and the length of ICU stays, it is of note that the majority of LND patients underwent extended surgery, such as hepatectomies, compared to more minor resections in nearly all patients of the LR group. Therefore, the available patient data could be more heterogeneous and ideal for a conclusive comparison regarding the morbidity associated with LND in patients with CRLM.

This study has several limitations. First of all, data were collected retrospectively from one centre only. Further, the included patient population is relatively small, and the LND and LR groups are quite heterogeneous regarding the size of the tumour and the extent of surgery. This is one of the main limitations of this study, as it makes a concluding comparison between the two groups difficult.

However, even with the LND group undergoing more extensive surgeries, no significant difference in overall complications or length of postoperative stay was seen. The rate between hepatectomies was only significantly higher in the LR group.

Furthermore, the LN+ group comprises only ten patients, allowing only limited conclusions about treating patients suffering from CRLM and positive perihilar LN. This topic needs further investigation.

Another possible confounder was the varying administration of chemotherapy in this patient cohort, which potentially influenced the oncological outcomes.

Altogether, this study was conducted to gain further insight into the outcomes of performing a hilar LND in our cohort, as this is still a controversial research topic. However, to make a more generalisable statement, further studies, especially randomised, multicentric, and standardised trials are needed with systematic LND and the administration of standardised chemotherapy regimens.

## 5. Conclusions

This study shows that preoperative imaging is not sensitive enough to detect affected LNs accurately and that CK22 staining helps identify micro-metastases and isolated tumour cells in resected LNs of patients with CRLM.

It also seems that perihilar LND is not associated with better recurrence-free or overall survival in patients with CRLM. Therefore, LND cannot be recommended as a routine procedure for patients with CRLM at present.

Whether perihilar LND will provide a survival benefit in the future through more accurate staging and thereby adjusted adjuvant treatment regimens needs further investigation.

## Figures and Tables

**Table 1:** Patient Characteristics.

Parameters	LND (*n* = 54)	LR (*n* = 58)	*p*-Value
Female Sex	26 (48.2)	19 (32.8)	0.124
Age, Years	63.5 [36–82]	64 [30–87]	0.457
Number of Performed Liver Resections			
First	43 (79.6)	42 (72.4)	0.388
Second	9 (16.7)	14 (24.1)	0.359
Third	2 (3.7)	2 (3.5)	>0.999
Obesity			
BMI, kg/m^2^	25 [15–38]	25 [17–36]	0.865
ASA Score			
II	1 (1.9)	5 (8.6)	0.208
III	52 (96.3)	52 (89.7)	0.274
IV	1 (1.9)	2 (1)	>0.999
Other Previous Liver Diseases			
Steatosis	23 (42.6)	20 (8.6)	0.439
Fibrosis	17 (31.5)	11 (19)	0.135
Previous Chemotherapy	38 (70.4)	35 (60.3)	0.323
Previous Surgery for CRC/CRLM	36 (66.7)	38 (65.5)	>0.999
Other Comorbidities			
Hepatitis B	1 (1.9)	3 (5.2)	0.619
Diabetes	4 (7.4)	5 (8.6)	>0.999
Cardiac History (CHD, MI)	1 (1.9)	7 (12.1)	0.062
Neurological History	7 (13)	10 (17.2)	0.604
COPD	2 (3.7)	4 (6.9)	0.680

Data are given as n (%) and median [min, max]. ASA: American Society of Anesthesiologists; BMI: body mass index; CHD: Coronary Heart Disease; COPD: Chronic Obstructive Pulmonary Disease; CRC: Colorectal Cancer; CRLM: Colorectal Liver Metastases, MI: Myocardial Infarct.

**Table 2:** Perioperative findings.

Parameters	LND (*n* = 54)	LR (*n* = 58)	*p*-Value
Size of Metastases, cm	3.07 [1–11]	2.1 [0.6–13]	0.002
<5 cm	45 (83.3)	51 (87.9)	0.592
≥5 cm	9 (16.7)	6 (10.3)	0.409
Number of Liver Metastases			
1	18 (33.3)	18 (31)	0.841
2	10 (18.5)	12 (20.7)	0.816
3	7 (13)	11 (19)	0.447
4	6 (11.1)	5 (8.6)	0.756
5	4 (7.4)	3 (5.2)	0.710
More Than 5	9 (16.7)	8 (13.8)	0.794
Surgical Technique	33 (61.1)	12 (20.7)	<0.0001
Anatomic Liver Resection	5 (9.3)	34 (58.6)	<0.0001
Non-Anatomic Liver Resection	16 (29.6)	10 (17.2)	0.179
Hepatectomies	34 (62.96)	8 (13.8)	<0.001
Right Hepatectomy	27 (50)	7 (12.1)	<0.0001
Left Hepatectomy	7 (13)	1 (1.7)	0.029
Operation Time, min	180 [79–395]	195 [99–726]	0.064
Blood Loss, mL	641 [50–2000]	222 [0–1600]	<0.0001

Data are given as *n* (%) and median [min, max].

**Table 3:** Postoperative Complications.

Parameters	LND (*n* = 54)	LR (*n* = 58)	*p*-Value
Overall Complications	46 (85.2)	48 (79.3)	0.800
None	8 (14.8)	10 (17.2)	0.800
I	15 (27.8)	20 (34.5)	0.542
II	9 (16.7)	10 (17.2)	>0.999
IIIa	7 (13)	3 (5.2)	0.192
IIIb	10 (18.5)	11 (9)	>0.999
IVa	3 (5.6)	1 (1.7)	0.351
IVb	0	2 (3.5)	0.496
V	2 (3.7)	1 (1.7)	0.608
Major Complications (CD > II)	22 (40.7)	18 (31)	0.0023
Complications within the first 30 days	34 (63)	31 (53.5)	0.342
Complications after 30 days	8 (14.8)	6 (10.3)	0.572
Length of ICU ^i^ stay, days	2 [1–14]	1 [1–35]	<0.0001
Length of postoperative stay, days	10 [3–47]	9 [3–55]	0.435

Data are given as *n* (%) and median [min, max]. ICU: Intensive Care Unit. ^i^ Refers to Clavien Dindo [23].

**Table 4:** Lymph Node Findings.

Parameters	LN+ (*n* = 10)	LN− (*n* = 44)	*p*-Value
Abnormal Perihilar LN on Preoperative Imaging (CT or MRI)			
Yes	2 (20)	1 (2.3)	0.085
No	8 (80)	43 (97.7)	0.085
Resected LN Stations (8,9,12)	4 (40)	14 (31.8)	0.715
Fewer Stations	0	7 (15.9)	0.326
More Stations	6 (60)	23 (52.3)	0.736
Number of Resected Perihilar and Extended LNs	5.5 [1–17]	7 [0–19]	0.411
Positive LN After Routine Pathological Examination	6 (11.1)		0.270 ^a^
Perihilar Micrometastasis (CK22 staining)			0.029 ^b^ 0.417 ^c^
>2 mm	6 (60)	0	
0.2–2 mm	3 (30)	0	
Single Tumour Cells	1 (10)	0	

Data are given as *n* (%) and median [min, max]. LN: Lymph Nodes. ^a^ Comparing abnormal perihilar LN on preoperative imaging and after routine pathological examination. ^b^ Comparing abnormal perihilar LN on preoperative imaging and after the use of CK22-staining. ^c^ Comparing abnormal perihilar LN on routine pathological examination and after the use of CK22-staining.

**Table 5:** Recurrence Rates and Overall Survival.

Parameters	LND (*n* = 54)	LR (*n* = 58)	*p*-Value
Follow-Up, Months	24 [0–49]	34.5 [0–130]	0.006
Additional Adjuvant Therapy			
Systemic	30 (55.6)	24 (41.4)	0.185
Local	28 (51.9)	10 (17.2)	<0.0001
Median RFS	10	15	0.076
6 months RFS	66.8%	74%	
1-year RFS	39.6%	54.6%	
2-year RFS	29.2%	41.8%	
Median Overall Survival, Months	49	60	0.959
6 Months Survival	90.4%	96.4%	
1-Year Survival	86.5%	90.6%	
2-Year Survival	77.9%	72.7%	

Data are given as *n* (%) and median [min, max]. RFS: Recurrence-free survival.

## Data Availability

The data supporting this study’s findings are available upon request from the corresponding author, due to privacy reasons as well as ethical restrictions, data cannot be available publicly.

## Abbreviations

ARDS	Acute Respiratory Distress Syndrome
ASA	American Society of Anaesthesiologists
BMI	Body Mass Index
CK22	Pancytokeratin Cocktail
COPD	Chronic obstructive pulmonary disease
CRC	Colorectal Cancer
CRLM	Colorectal Liver Metastases
ICU	Intensive Care Unit
LN	Lymph node
LND	Lymph node dissection
LR	Liver Resection only group
OS	Overall Survival
RFS	Recurrence-Free Survival
